# Potential biomarker identification for Friedreich’s ataxia using overlapping gene expression patterns in patient cells and mouse dorsal root ganglion

**DOI:** 10.1371/journal.pone.0223209

**Published:** 2019-10-30

**Authors:** Marissa Z. McMackin, Blythe Durbin-Johnson, Marek Napierala, Jill S. Napierala, Luis Ruiz, Eleonora Napoli, Susan Perlman, Cecilia Giulivi, Gino A. Cortopassi

**Affiliations:** 1 Department of Molecular Biosciences, University of California, Davis, Davis, California, United States of America; 2 Bioinformatics, University of California, Davis, Davis, California, United States of America; 3 Department of Biochemistry and Molecular Genetics, University of Alabama at Birmingham, Birmingham, Alabama, United States of America; 4 Department of Neurology, University of California, Los Angeles, Los Angeles, California, United States of America; Universidade de Sao Paulo Instituto de Biociencias, BRAZIL

## Abstract

Friedreich’s ataxia (FA) is a neurodegenerative disease with no approved therapy that is the result of frataxin deficiency. The identification of human FA blood biomarkers related to disease severity and neuro-pathomechanism could support clinical trials of drug efficacy. To try to identify human biomarkers of neuro-pathomechanistic relevance, we compared the overlapping gene expression changes of primary blood and skin cells of FA patients with changes in the Dorsal Root Ganglion (DRG) of the KIKO FA mouse model. As DRG is the primary site of neurodegeneration in FA, our goal was to identify which changes in blood and skin of FA patients provide a 'window' into the FA neuropathomechanism inside the nervous system. In addition, gene expression in frataxin-deficient neuroglial cells and FA mouse hearts were compared for a total of 5 data sets. The overlap of these changes strongly supports mitochondrial changes, apoptosis and alterations of selenium metabolism. Consistent biomarkers were observed, including three genes of mitochondrial stress (MTIF2, ENO2), apoptosis (DDIT3/CHOP), oxidative stress (PREX1), and selenometabolism (SEPW1). These results prompted our investigation of the GPX1 activity as a marker of selenium and oxidative stress, in which we observed a significant change in FA patients. We believe these lead biomarkers that could be assayed in FA patient blood as indicators of disease severity and progression, and also support the involvement of mitochondria, apoptosis and selenium in the neurodegenerative process.

## Introduction

Friedreich’s ataxia (FA) is a lethal neurodegenerative disease with a pediatric onset, and the most frequently occurring autosomal recessive inherited ataxia. FA is caused by a trinucleotide (GAAn) repeat expansion in intron 1 of the nuclear encoded gene frataxin (FXN) [1], which results in a 90% decrease in mitochondrial frataxin’s gene expression [2]. The lower the frataxin level, the earlier the age of onset and the worse the severity of the disease [3]. Frataxin protein is thought to be important for Fe-S cluster biogenesis and heme synthesis [4], thus supporting metabolically active cells through mitochondrial functions. However how this Fe-S defect leads to neurodegeneration is less clear and could potentially be addressed through high-dimensional gene expression analysis as in the current study.

The primary site of neurodegeneration in FA patients is the dorsal root ganglion (DRG), and progressive neural tissue damage ascends the dorsal spine including the spinocerebellar tract to degenerate the cerebellum and leads to a loss of voluntary motor function [5, 6]. Results of autopsies from FA patients have clearly indicated that the DRGs are significantly implicated in the progressive pathology of the disease [7]. Because DRG tissue is unavailable from living patients, finding overlap of blood-based or skin-based human FA biomarkers with the FA pathoneurophysiological process could be important for understanding the disease progression. Accessible peripheral cells could provide a window into the neuropathophysiological process inside the nervous system.

The first symptom of the disease is usually motor coordination loss in lower extremities [8] making the lumbar spinal tissue of particular interest. Adolescence is the average age of onset in FA, when symptoms present most often with lower extremity loss of coordination [9]. By choosing to analyze gene expression changes in the mid-lumbar spinal cord mouse DRGs, we assessed the target tissues innervating the lower extremities where clinical signs first present in humans [10].

Although there is support that frataxin defects affects iron-sulfur biogenesis, how this leads to DRG neurodegeneration is not clear, so there is still urgent need to understand pathomechanism as well as investigate therapeutic avenues for patients. The KIKO mouse model is an established FA model that results in reduced FXN transcription as well as reduced frataxin protein expression [11]. The neurobehavioral phenotype of these mice has also recently been clarified by our lab [12]. There is a defect in mitochondrial biogenesis in KIKO mice which also occurs in FA patient skin and white blood cells [13].

Using adolescent KIKO mice we isolated the primary target tissue of lumbar (L1-L4) DRG and spinal cord, and conducted an RNA sequencing experiment with WT and KIKO littermates. The DRG transcriptome genes were then correlated to FXN gene expression within each sample because FXN gene expression is associated with disease severity [14, 15]. The genes that were found to be significantly correlated with FXN expression in the mouse DRG tissues were then compared collaboratively with independent data sets from patient samples for comparative gene expression profiles in FA human tissues.

Lymphocytes were collected from patient blood and were analyzed for gene expression changes in a comparison of control, carrier, and patient cells [16]. Gene expression data was collected on fibroblasts derived from FA patient or unaffected control skin biopsies [17] in a comparison of FA to control [18]. For additional independent confirmation of frataxin-dependent gene expression changes mouse glial and mouse heart array data were compared as fourth and fifth data sets for confirmation of genes likely to be significantly altered in FA.

There was substantial overlap in significantly altered gene expression across the five data sets. Those genes that overlapped in greater than 3 data sets were evaluated for their relative strength as potential biomarkers of FA. The list of overlapping altered genes was also analyzed for their functional association to each other using STRING interaction network mapping and resulted in a distinct groups of antioxidant genes, apoptosis regulation, translation regulation, and mitochondrial genes.

## Materials and methods

### Mouse DRG

Mice were bred in the UC Davis laboratory colony until tissue extraction at 4 months of age. All research involving mouse tissues has been approved by The Institutional Animal Care and Use Committee (IACUC) protocol #18070. Mice were maintained on a (12h light/12hr dark cycle), and given food and *water ad libitum*. A total of 7 knock-in knock-out (KIKO) mice (C57BL6/j; fxn^GAA230/-), and 10 littermate wild type mice were used for these data. Mice were euthanized using inhaled isoflurane, followed by cervical dislocation before rapid tissue removal and storage in RNA*later*, Vilnius, Lithuania). Mouse DRG tissue was removed using a modified protocol for dorsal approach DRG removal of the lumbar spinal cord [19]. Total RNA was extracted from DRG lumbar tissue lysate using RNeasy Plus Mini Kit (Qiagen, Valencia, CA) following manufacturer’s instructions. The mRNA quantity was measured by NanoDrop 2000c Spectrophotometer (Thermo Scientific, Waltham, MA), and quality was measured by the 2100 Bioanalyzer using the Agilent RNA 6000 Nano Kit (Agilent Technologies).

### RNA Sequencing

The DNA Technologies Core prepared barcoded mRNA-Seq libraries for each sample, pooled them, and ran the pool on one lane of HiSeq (PE100). After evaluation of the first lane for quality and number of reads per sample, additional lanes were run to increase the number of reads (or pairs of reads). For RNAseq studies in vertebrates, 10 million fragments should detect ~80% of annotated genes, while 30 million fragments should detect ~90% of annotated genes (ENCODE Consortium Standards, Guidelines and Best Practices for RNA-Seq, V1.0, June 2011;[20]). Illumina read quality assessment was performed using FastQC. Scythe and Sickle were used for Illumina adapter and quality trimming. Trimmed reads were aligned to the Mus musculus mm10 genome using Tophat2 [21]. Cufflinks2 [22] were used on the paired-end reads to identify potential novel splice variants. The raw counts were derived from the alignments using the HTSeq-count python script [23]. Statistical analyses (tests of differential expression and tests of correlation were conducted using EdgeR or Limma-Voom).

Genes with expression less than 0.2 counts per million reads were filtered prior to analysis, leaving 20,444 genes. Analyses of the correlation of frataxin expression with the expression of other genes were conducted by fitting linear regression models using the Limma-Voom Bioconductor pipeline. Raw p-value was calculated for the test that the logFC for frataxin is different from 0. Benjamini-Hochberg false discovery rate (FDR) is used to calculate the adjusted P-value.

### Lymphocytes

The second independent set of data was derived from human blood samples collected from patients, carriers, and control individuals in a clinical setting at The UCLA Department of Neurology Program in Neurogenetics, and the UCLA Ataxia Center. Blood samples were collected from ~760 different individuals over ~ 5 years and results were also correlated to GAA repeat length to analyze peripheral gene expression data from patients with FA. All raw gene expression data is available for download in NCBI Gene Expression Omnibus (https://www.ncbi.nlm.nih.gov/gds) under accession number GSE102008.

All research involving patient/patient lymphocyte tissues was been approved by the Institutional Review Boards (IRB) protocol IRB#10–000833. The lymphocytes gene expression data was overlapped with the KIKO DRG gene expression data in search of commonly altered genes.

### Fibroblast

The third separate data set was generated from RNA sequencing reactions performed on 18 FA patient and 17 control fibroblast cell lines [17]. Fibroblast repositories were created from more than 50 patient skin biopsies, and numerous controls, and transformed in to induced pluripotent stem cells (iPSCs) All research involving patient/patient tissues has been approved by the Institutional Review Boards (IRB) at The University of Alabama at Birmingham (IRB Protocols: N160923005 and N160922011) and Children’s Hospital of Philadelphia (IRB Protocol 10–007864). The fibroblast gene expression results were then analyzed for overlap with mouse DRG transcriptome results.

### Mouse heart, glia

The fourth and fifth data sets were included as microarrays of Glial knockdowns of frataxin, and microarrays of mouse hearts lacking frataxin. Reverse transfection involving simultaneously transfecting and plating cells was performed using lipofectamine 2000 according to the manufacture protocol (Invitrogen). Briefly, transfection mix including siRNA (30nM final concentration, except 40nM for ND7/23) and transfection reagent was made and added to the wells in 6-well plates. The cells were harvested by trypsinization and about 0.3 million cells were plated in the wells with the transfection mix. siRNAs used in the study include: human frataxin siRNA described previously[20], AAC GUG GCC UCA ACC AGA UUU, and scrambled siRNA, CAG UCG CGU UUG CGA CUG GdTdT, human frataxin siRNA (ON-TARGETplus duplex #J-006691-07) and non-targeting siRNA (D-001810-0X), Rat frataxin siRNA (ON-TARGETplus SMARTpool, L-104901-01), mouse frataxin siRNA (ON-TARGETplus SMARTpool, #L-045500-00), and control siRNA (Non-Targeting Pool, #D-001810-10) from Dharmacon [24, 25]. Data was analyzed individually for each sample type using dChip v1.2. Within each group, samples were normalized to the median intensity chip and fluorescence values were generated using the perfect match-only model. Probe sets with a pCall > 40% and P < .05 were considered significantly altered. FDR testing was not performed.

### GPX activity

Glutathione peroxidase activity in FA patient whole blood was evaluated by using a commercially available kit from Abcam (#ab102530) and according to the manufacturer’s protocol. Hemoglobin was evaluated by using the ferricyanide-cyanide reagent. Results were expressed as IU per g Hb.

### Data analysis

All experiments were analyzed as a log fold change of FA cells compared to control, except for the mouse DRGs. Mouse DRGs results were analyzed as a regression analysis function of FXN expression. LogFC of DRG samples represents the change of a gene compared to FXN (+ r value represents the increase in a gene as FXN also increases). String Association Network Version 10.0 [26] tools were applied to overlap analysis of the 87 genes that were significant in 4/5 datasets. All settings were automated to default selections including a minimum required interaction score of 0.400, no clustering parameters specified, and with active interaction sources including: Textmining, Experiments, Databases, Co-expression, Neighborhood, Gene Fusion, and Co-occurrence. Network nodes represent all of “the proteins produced by a single protein-coding gene locus”. Edges represent protein-protein associations, “where proteins jointly contribute to a shared function” to determine the interactions shown. Further clarification of clusters associations and gene pathways were analyzed using DAVID Bioinformatics Resources [27], and using ENRICHR Comprehensive Gene Set Enrichment Analysis [28].

## Results

**Fig 1:**
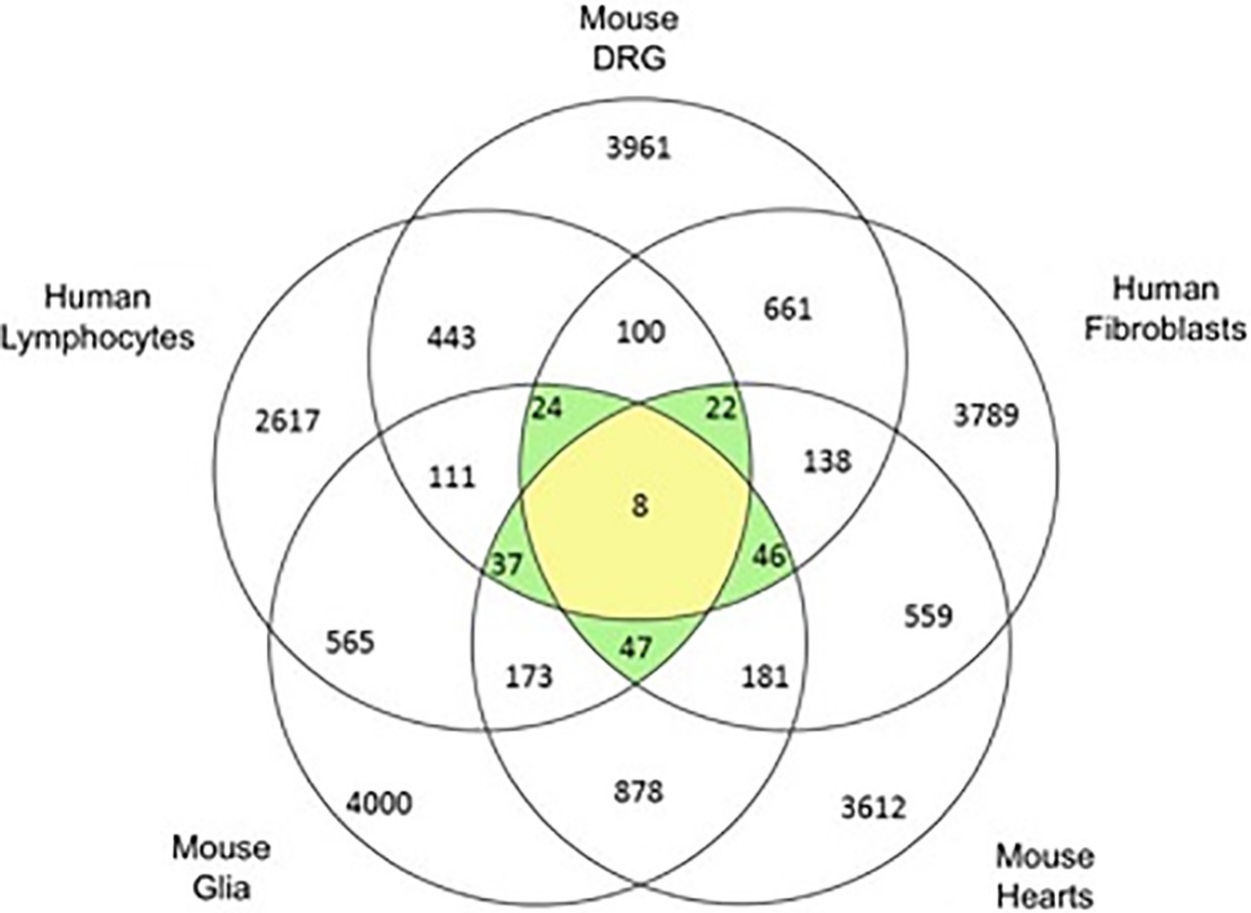
The overlap between human FA fibroblasts, human FA lymphocytes, KIKO mouse DRG, KIKO mouse heart and KIKO mouse glia cells (p < .05). Genes overlapping in 4/5 analyses passed to the next level of analysis.

**There is substantial overlap of gene profile in frataxin-deficient cells and tissues**. In the DRG transcriptome sequencing experiment of KIKO mice, 3961 genes were significantly correlated with FXN expression (FDR<0.05). In the human lymphocyte data set 2617 genes were significantly different in the comparison of patient to carriers (p<0.05). When DRG and lymphocyte data sets were compared to find common genes, 443 genes were found to be linked to frataxin expression on both lists. Next we incorporated expression data from FA patient fibroblasts, and found that 100 genes overlapped between the 3 experiments (Fig 1). We also tested the overlap of genes affected by frataxin depletion in FA mouse heart and FA glial cells (Fig 1). 87 most 'frataxin responsive' or 'consistently altered' transcripts differentially expressed in at least 4 experiments were identified (S1 Table).

**Fig 2:**
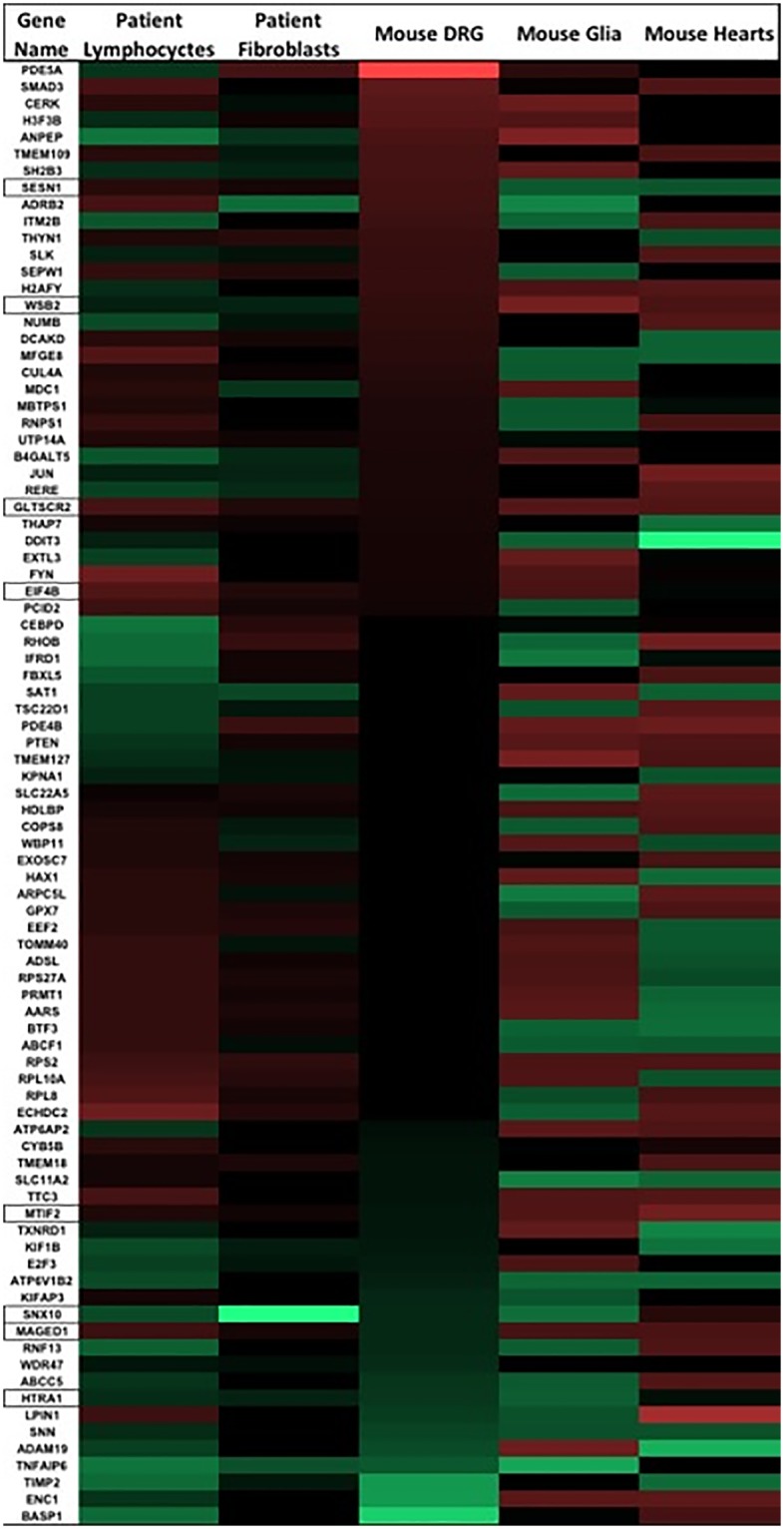
The 87 genes that were significantly changed in 4/5 experiments are shown as a heat map (Red = negative value, green = positive value, black = not significant). Lymphocyctes, fibroblasts, glia, and heart samples are all expressed as FA/Control. Mouse DRGs are analyzed as a FXN correlation, (-1)x logFC of gene/FXN expression. Outlined genes in the gene column were significant in all 5 datasets.

Although there was substantial overlap both of gene expression changes, and sometimes of the direction of change, there are also cases in which transcript direction was different in patients vs. controls in different tissues. For the remainder of our analysis we disregarded those differences, picking those 87 genes as the most 'frataxin-responsive', irrespective of sign change positive or negative in the patient vs. control comparison (Fig 2 red = negative value, green = positive value).

**Fig 3:**
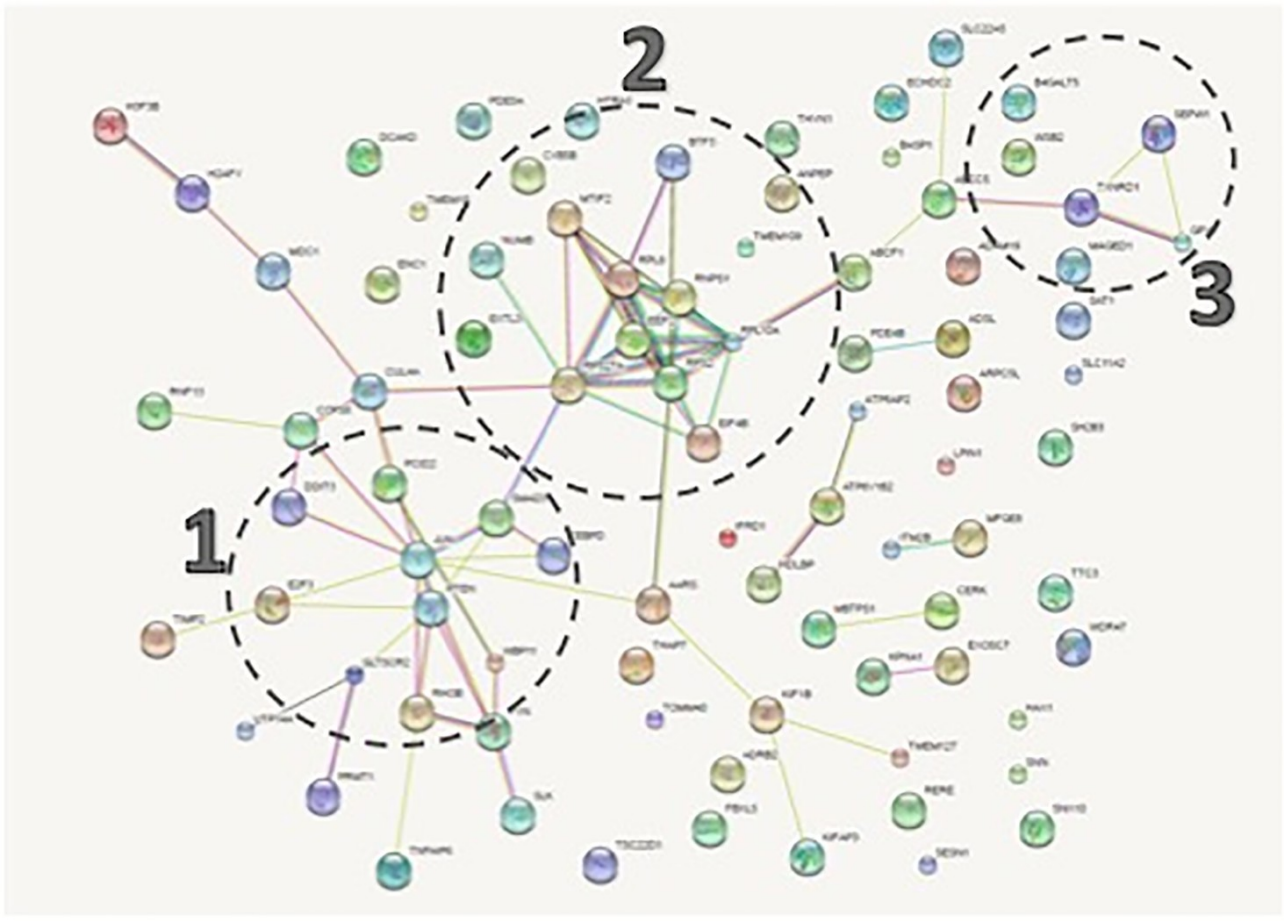
STRING network analysis of the 87 genes showing 3 clusters. Cluster 1 is primarily composed of the 10 genes significantly associated with regulation of apoptosis (FDR = .03). Cluster 2 is primarily composed of RNA transcription and mitochondrial translation related genes. Cluster 3 is primarily composed of selenium and glutathione metabolism genes.

**Fig 4:**
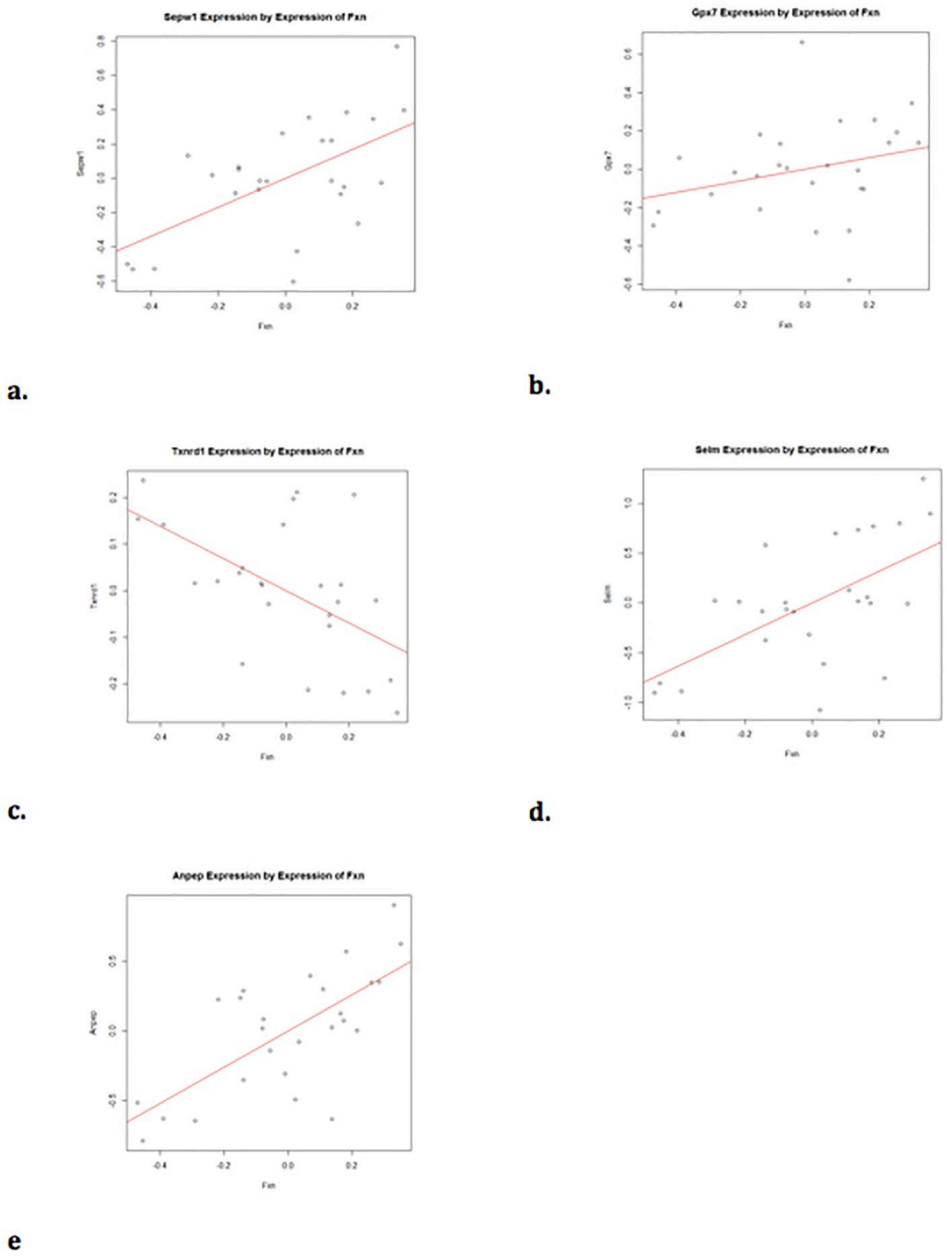
Cluster 3 genes include SEPW1, GPX7, and TXNRD1 which are significantly altered in 4/5 experiments (p<0.05). Fig 4a. In the mouse DRG SEPW1 expression is significantly correlated with FXN expression (r = 0.59) (FDR = 0.035). Fig 4b. GPX7 is not significantly correlated with FXN expression in mouse DRG (r = 0.28, FDR = 0.33). Fig 4c. TXNRD1 is significantly correlated with FXN expression in mouse DRG (r = -0.55, FDR = 0.04). Fig 4d. SELM is significantly correlated with FXN expression in mouse DRG (r = 0.6, FDR = 0.035). Fig 4e. ANPEP is significantly correlated with FXN expression (r = 0.69, FDR = 0.035).

**Fig 5:**
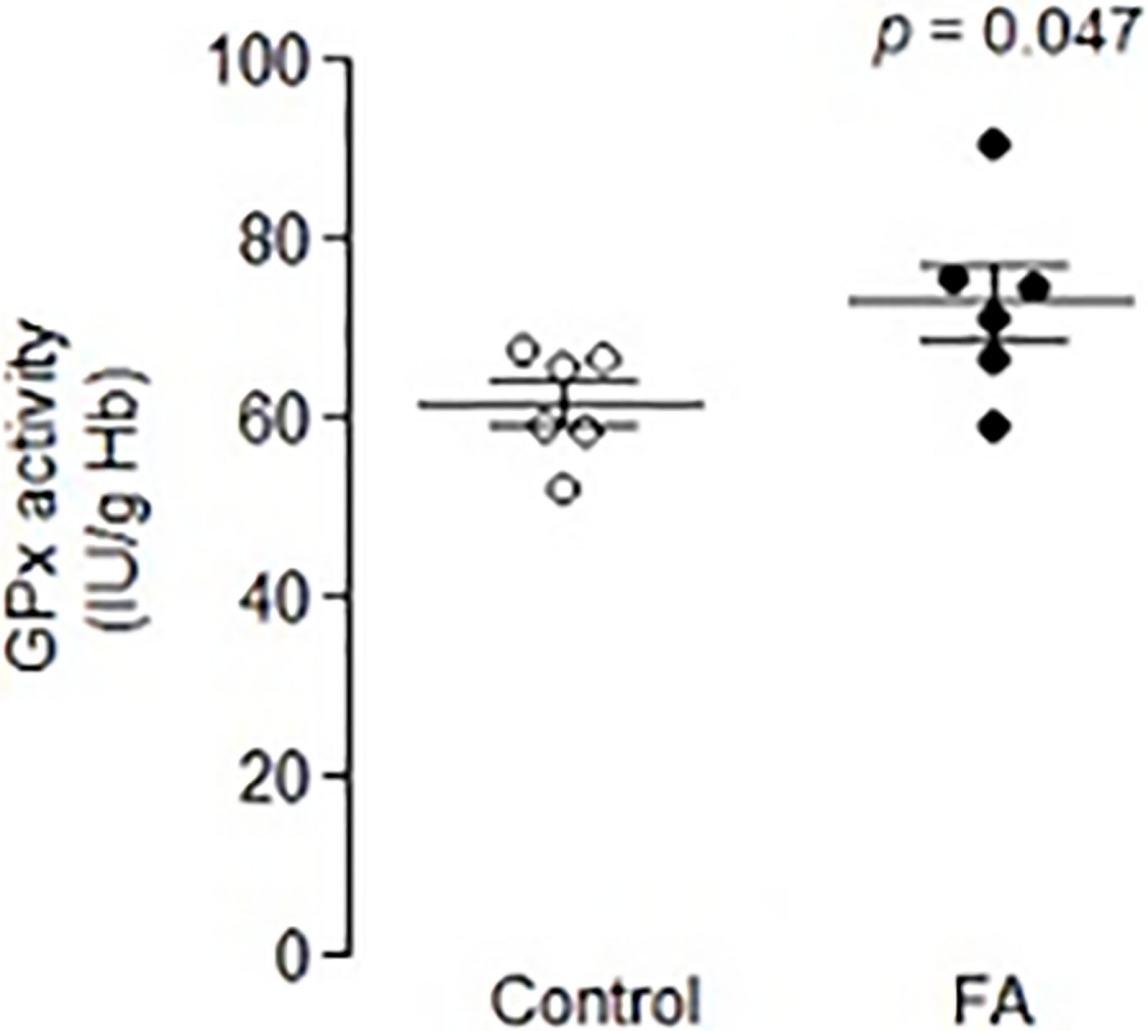
Glutathione peroxidase activity in whole blood was significantly increased in FA patients compared to controls (p = .047).

**STRING network analysis suggests multiple functional clusters**. We then used STRING Network Association version 10.0 [26] to analyze the 87 frataxin-responsive genes. Three distinct clusters emerged (Fig 3). The functional enrichment results for our network (p-value = 0.00783) included biological processes and cellular components (FDR < .05) The three distinct clusters were: biological processes related to apoptosis regulation in Cluster 1, mitochondrial translation and transcription in Cluster 2, and antioxidant genes in Cluster 3 (Fig 4). 18 of the 87 shared genes were mitochondrial. The STRING Association Network Cellular Compartment mitochondrial genes are: MTIF2, TOMM40, TXNRD1, LPIN1, PTEN, FYN, KIF1B, CERK, RPL10A, ADSL, DCAKD, CYB5B, ECHDC2, SEPW1, SLC11A2, TTC3, HAX1, and SNN (FDR = 0.01) (Fig 5). Thus 20% of genes altered in frataxin-deficient cells across 4 tissues are mitochondrial. Frataxin is a nuclear-encoded gene that is translated on cytosolic ribosomes and translocated to mitochondria [15]. One interesting gene from the mitochondrial cluster is Translocase of Outer Mitochondrial Membrane 40 (TOMM40). TOMM40 was significantly lower in patient lymphocytes as compared to carriers, and in frataxin-knock down glial cells. These data imply that frataxin deficiency causes many mitochondrial changes, and that mitochondrial biomarkers are altered in multiple FA patient tissues, and multiple frataxin-deficient cell lines. The results on (mitochondrial) translation and mitochondria in general suggest a fundamental involvement of mitochondrial biogenesis and metabolism in Friedreich’s, which has recently been confirmed by identifying a mitochondrial biogenesis defect in FA mouse model KIKO, patient cells and patient blood [13]

**The composition of cluster 1 includes 10 genes from the biological process pathway of Positive Regulation of Apoptotic Process** (FDR = 0.0324). Genes that induce apoptosis also scored highly, they include DDIT3, JUN, PTEN, SMAD3, RHOB, SLC11A2, ADRB2, TSC22D1, RPS27A, and RNPS1. These 10 apoptosis related genes could potentially play a role downstream of frataxin loss and upstream of neurodegeneration. A predisposition to apoptosis in FA (Wong et al., 1999) may be induced as a result of the intrinsic pathway of apoptosis due to DNA damage [29]. Notably among the cluster 1 genes, DNA Damage Inducible Transcript 3 (DDIT3 = CHOP = GADD153) is significantly upregulated in 4/5 of the experiments (S3b Fig), and significantly correlated with FXN expression in KIKO mouse DRG (r = 0.56). Stimulation of DDIT3/CHOP/GADD153 stress response [30–33] has been shown to occur as the immediate result of mitochondrial inhibition and the mitochondrial stress response [30, 31, 34]. Thus DDIT3/CHOP shows great promise as a biomarker of the mitochondrial stress that occurs in the pathophysiological mechanism of FA. DDIT3/CHOP induction also occurs as a result of cellular stress, and multiple genes in our Cluster 2 results relate to translational stress.

**The genes in cluster 2 are composed of the genes related to mitochondrial translation and transcription**. They are translation-related genes MTIF2 (involved in mitochondrial translation), EIF4b, EEF2, RPL8, RPS27A, RPS2, and RPL10A; and transcription genes BTF3, and RNPS1 (Enrichr). In fact MTIF2 was conserved in 5/5 overlapping datasets, supporting a defect/alteration in mitochondrial protein translation (Fig 2, S1a and S1b Fig). We recently demonstrated a defect in mitochondrial biogenesis in Friedreich’s ataxia in FA cells, FA KIKO mouse tissues, and FA human patients[13], which could result from this consistent defect in MTIF, involved in mitochondrial protein translation. Eukaryotic Translation Initiation Factor 4b (EIF4B) are cluster 2 genes that are significantly altered in all 5 data sets (S1c Fig). The cluster is also composed of RPS27A and RPS2, which encode for ribosomal protein components of the 40s ribosomal subunit. RPL10A and RPL8 encode for ribosomal 60s subunit components. EEF2 promotes the GTP-dependent translocation of the nascent protein chain from the A-site to the P-site of the ribosome [35]. BTF3 basic transcription factor 3 is required for transcriptional initiation. RNPS1 detects incomplete translation as an mRNA nuclear transport and mRNA surveillance protein, detecting truncated mRNA and initiates nonsense-mediated mRNA decay. Thus these high dimensional studies suggest a (mitochondrial) protein translation defect, and in fact other studies have recently confirmed a mitochondrial biogenesis defect in multiple FA mouse and human tissues.

**The genes in cluster 3 are primarily antioxidant genes related to selenium and glutathione**. Multiple antioxidant response genes altered are related to selenium metabolism and glutathione peroxidase activity: SEPW1, GPX7, and TXNRD1 (Fig 4), and if one reduces the stringency to 3/5 experiments, selenoprotein M (SELM) was also significant (p<0.05). SEPW1 and SELM are both biomarkers of bioavailable selenium [36–42], and both genes are down regulated when FXN is reduced in the DRGs. Because selenocysteine is at the active site of many selenium-dependent antioxidant enzymes, deficiency in selenium results in reduced antioxidant activity, including glutathione peroxidase activity [43–45], and the gene expression changes observed in our data may be important to understanding the involvement of frataxin as an antioxidant. Alanyl aminopeptidase (ANPEP) has been shown to positively regulate glutathione synthase [46], and its expression was also altered in 4/5 of the experiments (Fig 4e). Thioredoxin reductase 1 (TXNRD1) is a selenoenzyme that protects against oxidative stress [47], [48] which was significant in 4/5 of the experiments. Thus, frataxin-deficiency is associated with multiple parameters related to oxidative stress and selenometabolism. If frataxin deficiency alters selenium bioavailability, then reduced selenium bioavailability could potentially decrease the selenium-dependent antioxidant functions of thioredoxin reductase and other glutathione peroxidases[49], underlying the intrinsic sensitivity of FA patient cells to oxidant stress [50, 51].

For direct confirmation of the glutathione peroxidase antioxidant pathway in frataxin-deficient cells, a 6x6 study of FA patient whole blood was compared to control whole blood samples. There was no difference between controls and patients in hemoglobin (mg/mL) (p = 0.400), but GPx activity was significantly increased (IU/g Hb) in patient cells (p = 0.047) (Fig 5).

**Search for overlapping genes with available blood tests**. Since the underlying goal of this work is to identify new biomarkers that could be used clinically, we decreased screening stringency to altered in 3/5 of the experiments, which increased the total gene list, and then we crossed this expanded gene list with clinical blood tests available and orderable at the Mayo clinic diagnostic testing labs. The genes altered in 3/5 experiments and also with an orderable blood test from Mayo include: ENO2, LPIN1, SLC22A5, ATP6AP2, PTEN, SMAD3, TMEM127, ADSL, AARS, HAX1, and KIF1B. ENO2 (Mayo Clinic), and lipin 1 (LPIN1) (S4a Fig), are potentially interesting in terms of clinical monitoring of FA disease progression. ENO2 is found in mature neurons, and is linked to brain iron accumulation associated neurodegeneration [52]. Lipin 1 (LPIN1) was significantly altered in 4/5 of the data sets. Abnormal LPIN1 is associated with metabolic syndromes, vacuole regulation, and diabetes [53, 54]. Integral aspects of FA patient pathologies include increased likelihood of developing diabetes, metabolic abnormalities, and abnormal vacuole accumulation in the DRGs.

## Discussion

Here we conducted differential expression analyses of clinical and preclinical models of Friedreich’s ataxia. Our results suggest defects in mitochondrial translation and selenium metabolism are part of the pathophysiological process that leads to apoptosis and neurodegeneration. Since these clusters were identified in both clinical and preclinical samples, we suggest that they represent promising targets for further pathomechanistic studies. These results suggest that decreased FXN may alter selenometabolism which could explain the translational deficits (cluster 2), and may decrease the activity of antioxidant selenoenzymes (cluster 3), increasing the burden of oxidative stress, and leading to increased apoptosis (cluster 1). Thus, our results further support a more specific formulation of the oxidative stress hypothesis for FA, which has long been hypothesized to be a key mechanism of FA [55].

**Transcription and Translation Cluster**: The apoptosis genes may connect cluster 1 to cluster 2. This implies that the regulation of cell death is inextricably linked to the transcription and translation changes seen in frataxin-deficient cells. The GAA repeat expansion in FXN causes R-Loop formation [56], which leads to transcriptional repression. Basic transcription factor 3 (BTF3), which was significantly altered in 4/5 of the experiments, is required for transcriptional initiation, and may be responding to the need for increased transcription of FXN. Additionally, RNPS1 was a significantly altered gene in 4/5 of the experiments. RNPSI is integral to mRNA nuclear transport and mRNA surveillance, which may be detecting truncated frataxin mRNA and initiating nonsense-mediated mRNA decay.

Expression of translation genes was also altered, suggesting that cellular compensation for frataxin deficiency has an impact on translation efficiency of mitochondrial and cytoplasmic translation. Most mitochondrial proteins are translated on the cytoplasmic ribosomes, including Frataxin and other Fe-S cluster proteins [57]. Our results included significantly altered cytoplasmic translation genes: EEF2, RPL8, RPS27A, RPS2, and RPL10A. MTIF2 is a mitochondrial gene that is altered in 5/5 of the experiments. MTIF2 transfers mt-mRNAs into the mitoribosome [58] and translocation of frataxin to the mitochondria appears to be integral to oxidative phosphorylation [59, 60].

### A selenium-dependent cluster of genes: Relationship to glutathione and oxidative stress and potential use as a biomarker

**Selenometabolism cluster**: Selenium metabolism is intimately related with oxidative stress, as many important antioxidants enzymes (thioredoxin reductase, glutathione peroxidase) require the abnormal amino acid selenocysteine in order to carry out their activities. Selenium is a constitutive component of glutathione peroxidase [61]. If frataxin deficiency reduces the bioavailability of selenocysteine, causing selenocysteine deficiency, then one might predict multiple consequences. First, since selenocysteine is a rare but used amino acid with its own selenocysteine-tRNA, one might expect translational defects, and translation was a major functional cluster (Fig 3). The deficiency of SEPW1 and SELM in FA patient blood is consistent with a deficiency of bioavailable selenocysteine in FA patients.

Second, a defect in selenocysteine bioavailability would be predicted to reduce the antioxidant activity of multiple antioxidant selenoenzymes, including TXNRD1 and GPX7 which were both significantly altered in 4/5 of the experiments. Glutathione peroxidases and thioredoxin have also previously been shown to be significantly altered in the blood samples of FA patients [62]. These selenoenzymes were also found to be reduced in DRG of another FA mouse model, YG8 [49]. In support of an alteration in selenometabolism in FA, we observed a significant increase in GPX1 activity (Fig 5). These data tend to support an alteration of selenometabolism, possibly GPX1 activity is induced in response to a deficiency in the antioxidant selenium.

Alterations in selenoenzymes have been reported by others, including Helveston et al, 1996 [63]. Altered bioavailability of selenium results in reduced glutathione peroxidase activity, which has selenocysteine at its active site [45, 64, 65]. A deficiency in bioavailable selenium or selenocysteine could be an important consequence of frataxin deficiency, because many selenoenzymes are antioxidants. Additionally, selenium supplementation has been shown to increase the viability of FA fibroblast’s viability [66]. SEPW1, and SELM to some extent, are biomarkers of selenium status [67], and TXNRD1, thioredoxin reductase 1, is a selenium containing enzyme. Many glutathione peroxidases require selenium [64], GPX7 does not [68]. The selenoenzymes that are thought to provide antioxidant protection against ROS induced cellular damage are GPX—GPX1, GPX3, GPX4, GPX5, and GPX6 [69], and there is substantial support for antioxidant deficiency in FA [34, 49, 70].

Several markers of selenium status and selenium-related enzymes were significantly overlapping in 3/5 experiments. Those have not been included in these results to remove speculation. However, animals deficient in selenium or vitamin E develop white muscle disease, a myodegenerative condition considered to result from defects in antioxidant activity [71, 72]. Selenoprotein W was altered in 4/5 experiments. Selenoprotein W has been shown by [73] Flohe, and Steinbrenner) as the most responsive blood biomarker to selenium status. Based on the observed data we propose that frataxin deficiency may be associated with a change in bioavailable selenium or selenocysteine, which results in a deficiency in selenoenzymes and an antioxidant enzyme deficiency. Selenium level in blood and serum is available to be ordered through Mayo Clinic laboratories.

**Apoptosis Regulation Cluster**: From the STRING analysis, multiple apoptosis regulation genes were altered. These are consistent with the idea that frataxin deficiency may cause oxidative stress to the cells, result in DNA damage, and increase sensitivity to apoptosis [50]. DDIT3 and PTEN are notable transcripts from our cluster 1 results. DDIT3 (also known as CHOP), which we observed to be significantly altered in 4/5 of our data has already been shown to be activated downstream of mitochondrial stress [4, 34]. DDIT3 has also been linked to global shut down type of cellular response to stress [32].

PTEN was significantly altered in 4/5 of the data sets. PTEN has been linked to PREX1 activation [74](S4c Fig). PREX1 = Phosphatidylinositol-3,4,5-Trisphosphate Dependent Rac Exchange Factor 1 was also significantly changed in 3/5 of the experiments. PREX1 has been shown to bind to Rac1, which is a NADPH oxidase [75], and we have previously observed PREX1 to be altered in a smaller screen for FA biomarkers [76]. PREX1 induces PI3K inhibition-induced apoptosis and also has potential as a stress response biomarker. PREX1 was significant in 3/5 of the experiments. PREX1 has been shown to bind to Rac1, which is a NADPH oxidase [75], and we have also previously observed PREX1 to be altered in a smaller screen for FA biomarkers [76].

**Other potential blood based biomarkers of FA that already exist as blood tests is ENO2**. We observed significantly altered expression of ENO2 in 3/5 experiments. Enolase 2 = Neuron-specific enolase is expressed in neurons and lymphocytes, and was one of the most altered genes in FA blood. It has also been shown that increased ENO2 corresponds to demyelination [77]. ENO2 is known to play a role in at least one other neuronal disease that results in motor coordination loss [78]. Recently, serum neuron specific enolase (NSE) levels have been reported as a biomarker for multiple sclerosis disease progression [79]. ENO2 is a clinically-run blood test (Mayo Clinic, Quest Diagnostics), and could be used to assess markers of mitochondrial function and/or myelination status in FA patients. If we relax constraints to 3/5 experiments and then cross these by blood tests available at Mayo clinic the list of blood tests include: ENO2, LPIN1, SLC22A5, ATP6AP2, PTEN, SMAD3, TMEM127, ADSL, AARS, HAX1, and KIF1B. Thus these are potential biomarkers to be explored in FA for which commercially available clinical tests exist.

**List of potential biomarkers with support from blood to mice**. Our results suggest a group of biomarkers to be tested in FA patients vs. controls, including these four seleno-related: SEPW1, SELM1, TXRD1, ANPEP, and the antioxidant-related PREX1. As a marker of apoptosis and mitochondrial defects DDIT3 also shows potential. ENO2 is currently available from clinical labs, was quite altered in blood, and shows promise. MTIF2 was a significant biomarker in 5/5 of the experiments and could potentially act to clarify ongoing mitochondrial changes as a result of disease progression or therapy.

## Conclusions

Overlapping patterns of gene expression in 5 independent frataxin-deficient tissues led to a limited set of 5 potential biomarkers. Cluster analysis identified a mitochondrial transcription translation cluster, and a selenium metabolism cluster, and an apoptotic cluster. Multiple genes that could be considered as biomarkers of frataxin deficiency in blood, that agreed with other tissues, were identified, and these included DDIT3, MTIF2, SEPW1, ENO2, and PREX1. Of these, blood tests for ENO2, Lpin1, Serum Selenium, and Blood Selenium are clinically available blood tests from Mayo Clinic laboratories, and thus could be included in new biomarker clinical trials for Friedreich’s. These 'most consistent' biomarkers appear to be ready for testing in humans, and could be tested in blood relative to repeat length, extent of frataxin deficiency, age of onset, and clinical assessment of motor score. Furthermore, these data suggest pathomechanistically that frataxin deficiency perhaps through selenium metabolism alters mitochondrial biogenesis and consequently triggers apoptosis and neurodegeneration.

## Supporting information

S1 Fig **a**. Cluster 2 genes include Mitochondrial Translational Initiation Factor 2 (MTIF2) which is significantly correlated with FXN expression (r = -0.69) (FDR = 0.036). **b**. MTIF2 expression is significantly altered in all 5 data experiments. **c**. Eukaryotic Translation Initiation Factor 4b (EIF4B) is significantly correlated with FXN expression in KIKO mouse DRG (r = 0.52) FDR = 0.046.
(TIF)

S2 Fig A substantial number of mitochondrial genes are significantly associated with FXN expression in 4/5 gene sets. String Association Network results shown with Cellular Compartment: Mitochondrion in red (FDR = 0.01) were manually clustered for this figure.
(TIF)

S3 Fig **a**. Cluster 1 genes regulating apoptosis include DNA damage inducible transcript 3 (DDIT3) which is correlated to FXN expression (r = 0.56) (FDR = 0.049). **b**. DDIT3 is significantly altered in FA patient lymphocytes, FA mouse glial cells, KIKO mouse DRG, and FA mouse heart (p<0.05).
(TIF)

S4 Fig **a**. LPIN1 expression is significantly correlated with FXN expression (r = -0.54) (FDR = 0.039). **b**. ENO2 expression is significantly correlated to FXN expression (r = -0.53) (FDR = 0.042) in mouse DRG. **c**. PREX1 is significantly correlated with FXN expression in mouse DRG (r = 0.54) (FDR = 0.041).
(TIF)

S1 Table The 87 genes that were significantly changed in 4/5 experiments are shown as raw values. (TIF)
