# Supplementary figures and images for: Potential biomarker identification for Friedreich’s ataxia using overlapping gene expression patterns in patient cells and mouse dorsal root ganglion
Source: PLoS One. 2019 Oct 30;14(10):e0223209. doi: 10.1371/journal.pone.0223209 (PMC6821053; doi:10.1371/journal.pone.0223209)

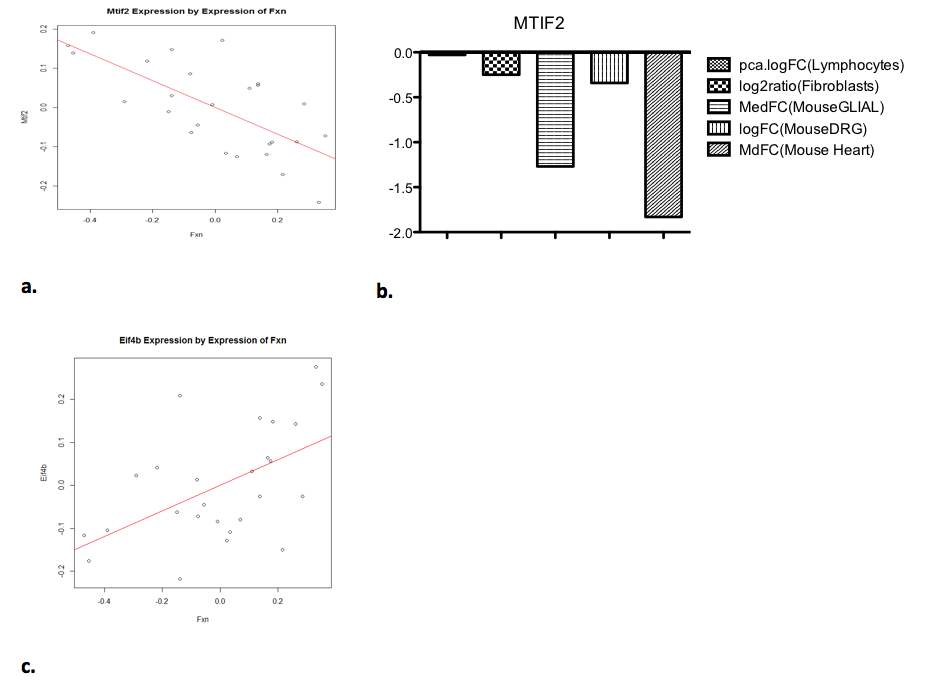

Supplement: S1 Fig — a. Cluster 2 genes include Mitochondrial Translational Initiation Factor 2 (MTIF2) which is significantly correlated with FXN expression (r = -0.69) (FDR = 0.036). b. MTIF2 expression is significantly altered in all 5 data experiments. c. Eukaryotic Translation Initiation Factor 4b (EIF4B) is significantly correlated with FXN expression in KIKO mouse DRG (r = 0.52) FDR = 0.046. (TIF) [file pone.0223209.s001.tif]

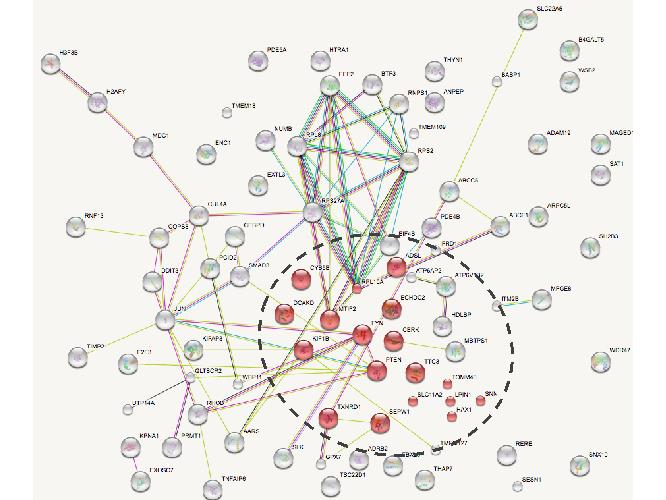

Supplement: S2 Fig — String Association Network results shown with Cellular Compartment: Mitochondrion in red (FDR = 0.01) were manually clustered for this figure. (TIF) [file pone.0223209.s002.tif]

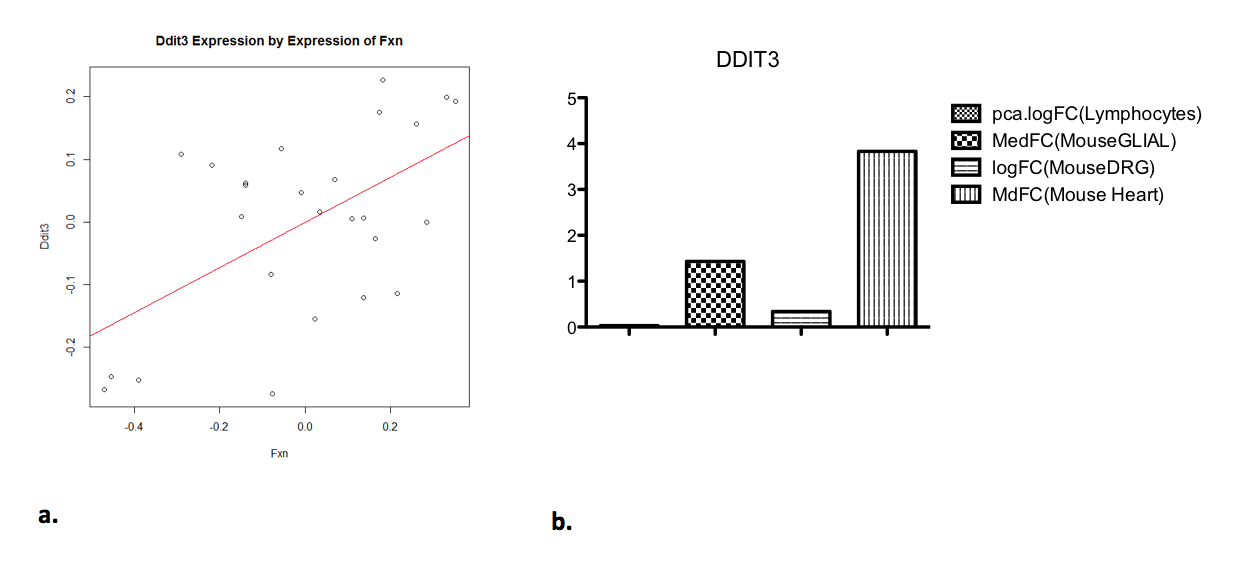

Supplement: S3 Fig — a. Cluster 1 genes regulating apoptosis include DNA damage inducible transcript 3 (DDIT3) which is correlated to FXN expression (r = 0.56) (FDR = 0.049). b. DDIT3 is significantly altered in FA patient lymphocytes, FA mouse glial cells, KIKO mouse DRG, and FA mouse heart (p<0.05). (TIF) [file pone.0223209.s003.tif]

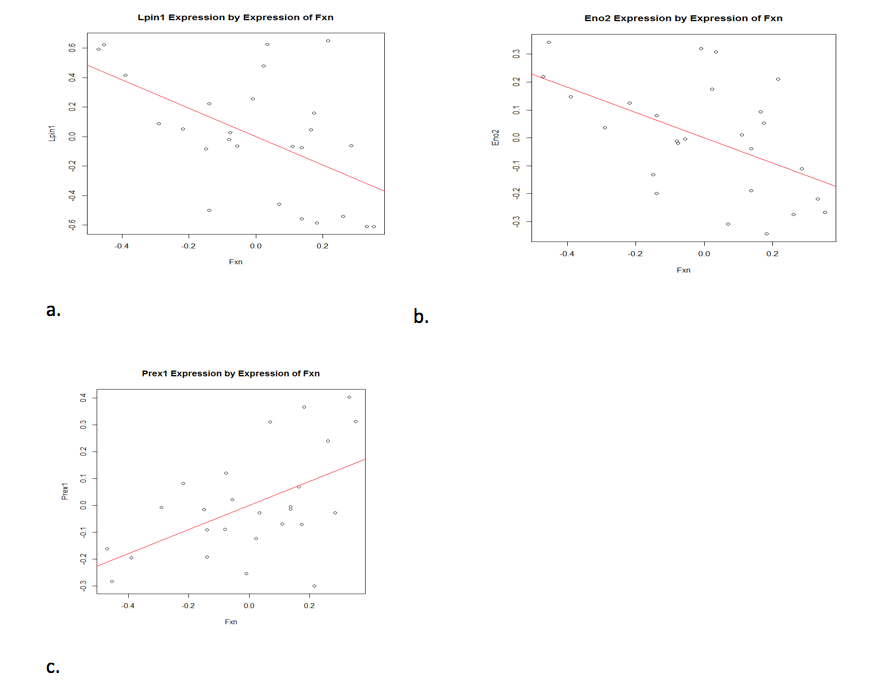

Supplement: S4 Fig — a. LPIN1 expression is significantly correlated with FXN expression (r = -0.54) (FDR = 0.039). b. ENO2 expression is significantly correlated to FXN expression (r = -0.53) (FDR = 0.042) in mouse DRG. c. PREX1 is significantly correlated with FXN expression in mouse DRG (r = 0.54) (FDR = 0.041). (TIF) [file pone.0223209.s004.tif]

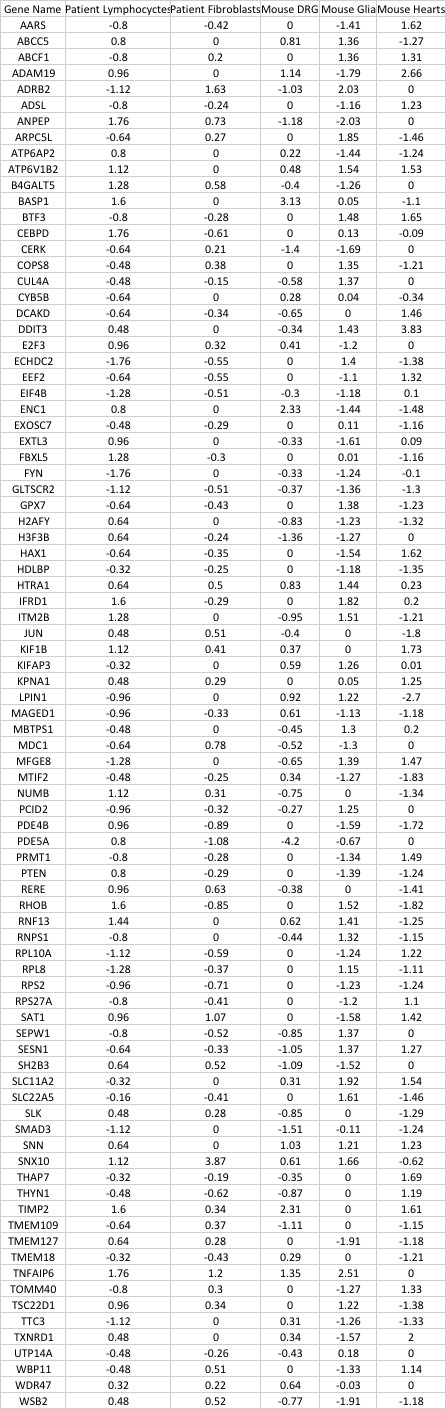

Supplement: S1 Table — (TIF) [file pone.0223209.s005.tif]
